# The implication of necroptosis-related lncRNAs in orchestrating immune infiltration and predicting therapeutic efficacy in colon adenocarcinoma: an integrated bioinformatic analysis with preliminarily experimental validation

**DOI:** 10.3389/fgene.2023.1170640

**Published:** 2023-08-02

**Authors:** Shizhe Li, Xiaotong Wang, Yajun Liu, Junbo Xiao, Jun Yi

**Affiliations:** ^1^ Xiangya Hospital, Central South University, Changsha, China; ^2^ Hunan Provincial People’s Hospital, Changsha, Hunan, China

**Keywords:** necroptosis-related genes and lncRNAs, colon adenocarcinoma, stemness, bioinformatic analysis and experimental validation, immune infiltration

## Abstract

**Background:** Necroptosis contributes significantly to colon adenocarcinoma (COAD). We aim to assess the relationship between immunoinfiltration and stemness in COAD patients through the development of a risk score profile using necroptosis-related long noncoding RNAs (NRLs).

**Methods:** Our study was based on gene expression data and relevant clinical information from The Cancer Genome Atlas (TCGA). Necroptosis-related genes (NRGs) were obtained from the Kyoto Encyclopedia of Genes and Genome (KEGG) database. Pearson correlation analysis, Cox regression, and least absolute shrinkage and selection operator (LASSO) regression were used to determine the NRL prognositic signature (NRLPS). NRLs expression was examined using qRT-PCR method. Several algorithms were used to identify relationships between immune cell infiltration and NRLPS risk scores. Further analysis of somatic mutations, tumor stemness index (TSI), and drug sensitivity were also explored.

**Results:** To construct NRLPS, 15 lncRNAs were investigated. Furthermore, NRLPS patients with high-risk subgroups had lower survival rates than that of patients with low-risk subgroups. Using GSEA analysis, NRL was found to be enriched in Notch, Hedgehog and Smoothened pathways. Immune infiltration analysis showed significant differences in CD8^+^ T cells, dendritic cell DCs, and CD4^+^ T cells between the two risk groups. In addition, our NRLPS showed a relevance with the regulation of tumor microenvironment, tumor mutation burden (TMB) and stemness. Finally, NRLPS demonstrated potential applications in predicting the efficacy of immunotherapy and chemotherapy in patients with COAD.

**Conclusion:** Based on NRLs, a prognostic model was developed for COAD patients that allows a personalized tailoring immunotherapy and chemotherapy to be tailored.

## Graphical Abstract

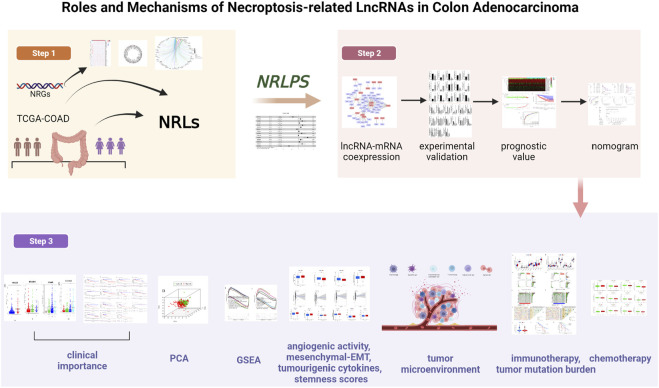

## Introduction

As a major cancer of the gastrointestinal tract, colorectal cancer (CRC) is among the most common types, accounting for 38.8% of gastrointestinal cancer cases (Arnold et al., 2020; Lu L. et al., 2021). As a combined cancer type of both sexes, it has the distinction of being the third most frequently diagnosed and the second deadliest in the United States (Siegel et al., 2020). Adenocarcinoma constitutes over 90% of CRCs (Lotfollahzadeh et al., 2022). Colon adenocarcinoma (COAD) is increasingly prevalent among patients with early-onset colorectal cancer (EOCRC) (Montminy et al., 2021). APC mutation is currently suggested to be one of the earliest initial events in CRC and drive the clinical phenotype related to infiltration and metastasis, which is linked to the sustained activation of the Wnt signaling pathway (Caspi et al., 2021). The Wnt signaling pathway is thought to facilitate the stemness of cancer stem cells (CSC), and alter the anti-tumor activity of immune cells (e.g., dendritic cells and T cells), causing tumor immune escape and therapeutic resistance (Goldsberry et al., 2019). Immunotherapy and targeted therapy are emerging as prominent treatment options that are expanding beyond chemotherapy; however, their effectiveness remains elusive, as their treatment responses and survival outcomes that are unpredictable (Brenner et al., 2014; Benson et al., 2021). Epidermal growth factor receptor (EGFR) inhibitors are currently the primary therapeutic option for metastatic colon cancer, due to the limitations of innate and acquired drug resistance mechanisms, which urgently require the guidance of predictive biomarkers (Martinelli et al., 2020). In addition, 5-year survival rates for patients with COAD are largely determined by stage. For stage I patients, the survival rate exceeds 90%, while stage IV metastatic patients have a dramatic decline to only 11% (Miller et al., 2022). The utilization of innovative and dependable biomarkers can facilitate a more comprehensive evaluation of disease progression and can provide a more personalized approach to treating patients.

Known as a regulated cell death mode with a caspase-dependent way, necroptosis induces inflammatory responses that differ from those induced by apoptosis during programmed cell death (PCD) (Bertheloot et al., 2021). There is a prevalence of necroptosis in many diseases, including chronic hepatitis (Mohammed et al., 2021), disease with neurodegenerative processes (Yuan et al., 2019), and Chronic obstructive pulmonary disease (COPD) (Lu Z. et al., 2021). Also, it has been proposed that necroptosis plays a dual role in the development of cancer (Qin et al., 2019). A malfunctioning apoptosis in tumor cells can result in necroptosis, which inhibits tumor metastasis and progression. Patients suffering from breast, ovarian, gastric, colon, and pancreatic cancer may face a poorer prognosis when mixed-lineagekinasedomain-like protein (MLKL), a necroptosis executor, is low in expression (Hu et al., 2018). And a key role for receptor-interacting serine/threonine-protein kinase 3 (RIPK3) in preventing hematopoietic malignancies is through specific mediation of necroptosis in myeloid leukaemia cells (Hockendorf et al., 2016). According to recent findings, damage-associated molecular patterns (DAMPs) produced by necroptosis cells are responsible for the development of dendritic cells in the tumor microenvironment (TME) and to the cross-initiation of CD8^+^ T cells within it (Tang et al., 2020), triggering antitumor immunity. Apart from this, tumor immunity and proliferation have also been reported to be involved in the necroptosis signaling pathway, suggesting that targeting necroptosis may be a viable option for new tumor therapies (Yatim et al., 2015; Bolik et al., 2022). It is speculated that necroptosis may alter the TME and promote the infiltration of tumor-infiltrating lymphocytes (TILs), which, in turn, may increase the response to immune checkpoint inhibitors (ICIs) administered to patients with advanced cancer (Rosenbaum et al., 2021). Hence, targeting the mechanism of necroptosis could, for the time being, be a promising treatment option. However, further study is warranted before we can fully understand the role that necroptosis plays in COAD and its impact on tumor immunity.

LncRNAs (long non-coding RNA) are transcripts with nucleotides greater than 200 that lack protein coding ability (Bhan and Mandal, 2014). Yet, they contribute to diverse biological functions, such as the regulation of chromatin formation and the integrity of the genome (Yao et al., 2019; Statello et al., 2021). In addition, it is important to note that multiple mechanisms are involved in the proliferation, invasion, and resistance to chemotherapy of COAD caused by lncRNAs (Chen and Shen, 2020; Liu et al., 2021), including angiogenesis and epithelial-mesenchymal transition (EMT) (Sun et al., 2019). Li et al. (2021) showed that lnc-RP11-536 K7.3 stimulated colon cancer proliferation and resistance to chemotherapeutics by mediating angiogenesis and glycolysis. According to Wang et al. (2020) HIF-1α induces MiR-205 to destabilize and degrade lncRNA HITT, contributing to the angiogenesis and growth of COAD. Further evidence shows PCD-related lncRNAs can help predict the prognosis of COAD patients and evaluate their treatment efficacy (Jiang et al., 2021; Yang et al., 2021). Evidence is mounting that necroptosis-associated lncRNA (NRLs) can be used to screen, diagnose, and prognosticate other tumors (Hu et al., 2022). It is worth noting, however, that the research demonstrating the use of NRLs for prognosis evaluation in COAD patients remains limited.

Through bioinformatics and statistical analysis, we have managed to conduct a comprehensive and in-depth analysis of the expression and interactions of NRLs in COAD patients with the purpose of highlighting the immunological aspects and examining the mechanisms of NRLs in the progression of COAD, whose expressions have been validated experimentally. Furthermore, we examined the potential application of this model in regulating the tumor immunity and stemness of patients with COAD as well as predicting their response to immunotherapy and chemotherapy, which enables us to develop new strategies for improving patient survival and selecting the appropriate treatment approach.

## Materials and methods

### Compilation of information

To investigate the clinical information and genetic characteristics of individuals suffering from COAD, 480 tumor samples and 41 normal samples were downloaded from the TCGA database (https://portal.gdc.cancer.gov). COAD somatic mutation datasets and copy number variation (CNV) data are also available from the TCGA and UCSC Xena websites. With the help of Ensembl Human Genome Browser (Cunningham et al., 2019), we classified lncRNAs and protein-coding genes (http://asia.ensembl.org/index.html). An overview of the clinical characteristics of patients with COAD is presented in Supplementary Material S1. There are data publicly available from TCGA, and the current study was conducted in compliance with TCGA data access policies and publication guidelines (http://cancergenome.nih.gov/abouttcga/policies/publicationguidelines).

### Molecular identification of necroptosis-associated genes and lncRNAs

There are 161 necroptosis-related genes (NRGs) listed in Supplementary Table S2, which is derived from the Kyoto Encyclopedia of Genes and Genomes (KEGG) database. In order to identify differentially expressed NRGs in TCGA tissues, we used the “limma” and “edgeR” R packages, displaying the results as heatmaps and filtering the data according to a |log fold change (FC)| > 2 and false discovery rate (FDR) < 0.05. The “maftools” package was used to generate mutation frequency and oncoplot waterfall plots of NRGs patients with COAD. With the use of the “RCircos” package in R, we were able to locate the CNV alteration of NRGs on 23 chromosomes. After that, Gene Ontology (GO) classifications, including biological processes (BP), cellular components (CC), and molecular functions (MF), were carried out using the “ggplot2” and“clusterProfiler”R package. To determine whether NRGs expression correlates with corresponding lncRNA expression, Pearson correlation coefficients were then calculated. We identified necroptosis-related lncRNAs (NRLs) based on the following thresholds: *p*-value <0.001 and correlation coefficient |R| > 0.3 (Supplementary Material S3).

### Incorporation of NRLs into a prognostic signature

The effect of NRLs on overall survival (OS) in patients with COAD was evaluated using univariate Cox regression analysis (*p* < 0.05) (Supplementary Material S4). Afterward, these NRLs were included in least absolute shrinkage and selection operator (LASSO) Cox regression analysis utilizing the “glmnet” R package and tenfold cross-validation (Friedman et al., 2010). We finally performed a multivariate Cox regression analysis to construct the optimal NRL prognostic signature (NRLPS).

Calculating the prognostic signature risk scores required the following formula:

Risk score = $\sum_{i=1}^{n}\beta_{i}$ ∗ (expression of lncRNA_i_), β means a regression coefficient.

According to the median values of the NRLPS risk scores, we divided the data into low- and high-risk groups. A Kaplan-Meier survival analysis was performed to assess our risk score’s survival rate. An independent prognostic prediction of the risk score was examined using univariate and multivariate Cox regression analyses. For the purposes of assessing the accuracy of the prediction and comparing the NRLPS with a variety of patients’ characteristics, the receiver operating characteristic (ROC) curves and the area under the curve (AUC) values were employed as predictors. A correlation analysis was conducted between the NRLPS risk scores and a number of clinical manifestations, as well as an analysis of survival between the NRLPS risk scores and the clinicopathological stratifications. Through the use of R package “survival” and “rms”, a nomogram based on different clinicopathological factors was generated to predict survival at 1, 3, and 5 years. In calibration graphs, the actual survival rates are compared with the nomogram-predicted survival rates, where the overlap with the reference line indicates the model’s accuracy.

### Culture of cell lines and COAD tissues

NCM460, HCT116, HT29, SW480, and CaCO2 cell lines were donated by Cancer Research Institute of Central South University (Hunan, China). They were maintained in DMEM or RPMI 1640(Hyclone, Logan, UT, United States) supplemented with 10% FBS,streptomycin (100 ug/mL) and penicillin (100 U/mL), and were cultured in incubators containing 5% CO2 at 37°C. Four pairs of COAD and adjacent non-tumor tissue samples were obtained from patients who underwent surgery at the Xiangya Hospital of Central South University (Hunan, China).

### Quantitative reverse transcription polymerase chain reaction (qRT-PCR)

Total RNA was extracted from the tissue samples and cells by TRIzol reagent (Invitrogen, Carlsbad, United States) according to the manufacturer’s protocol. Then cDNA was synthesized using the Reverse Transcription Kit (TransGen Biotech, Beijing, China). qPCR assay was conducted using SYBR-Green PCR Master Mix (TransGen Biotech, Beijing,China) under conditions including 30s at 94°C and 40 cycles of 5s at 94°C, 15s at 60°C, and 10s at 72°C. The sequences of the primers and results used in this study are listed in Supplementary Material S5.

### The establishment of a co-expression network between necroptosis-related lncRNAs and mRNAs

Through the usage of the Cytoscape software (http://www.cytoscape.org/), the co-expression network of mRNAs and lncRNAs was generated to visualize the correlation between the NRLs and corresponding mRNAs. A Sankey diagram was then constructed using R’s “ggalluvial” package to further illustrate the degree of correlation between NRLs (risk/protective) and their related mRNAs.

### Principal component analysis (PCA)

In analyses of the whole genome, lncRNAs, NRLs, and NRLPS, PCA was utilized through “scatterplot3D″ R package to uncover patterns and interpret exploratory visualizations.

### Gene set enrichment analysis (GSEA)

GSEA was performed to identify the potential signaling pathways implicated in the occurrence and progression of patients with COAD as mediated by NRLPS, including GO and KEGG, which were enriched in different NRLPS risk groups (Subramanian et al., 2005). *p* < 0.05 and false discovery rate (FDR) q‐value <0.05 were statistically significant.

### An analysis of the immunogenomic landscape

We compared different algorithmic approaches, including CIBERSORT (Newman et al., 2015; Charoentong et al., 2017), CIBERSORT -ABS (Tamminga et al., 2020), QUANTISEQ (Finotello et al., 2019; Plattner et al., 2020), EPIC (Racle et al., 2017), ESTIMATE (Yoshihara et al., 2013), MCPcounter (Shi et al., 2020), single-sample gene set enrichment analysis (ssGSEA) (Yi et al., 2020) and TIMER (Li et al., 2017), in an attempt to comprehensively analyze the immune differences between high-risk and low-risk groups of the TCGA cohort.

### Differential expression of immune checkpoint genes (ICGs) and MHC molecules in high-/low-risk groups

It is currently acknowledged that ICGs are a promising treatment option for COAD (Emambux et al., 2018). It was decided to evaluate the differences in the levels of expression of selected ICGs and MHC molecules in high-risk and low-risk groups to provide guidance for the selection of treatment that may be most appropriate for each individual.

### Tumor-related scores and tumor stemness indices (TSIs) analysis

Several tumor-related scores of each tumor samples were calculated by ssGSEA algorithm, including tumourigenic cytokines, EMT, angiogenic activity and stemness scores.

There was reported to be a correlation between TSIs and active biological processes in stem cells as well as a greater degree of tumor dedifferentiation based on the results of a previous study (Malta et al., 2018), including mRNAsi, EREG-mRNAsi, mDNAsi, EREG-mDNAsi, and ENHsi. An oxidative stress-related gene list was obtained from the GeneCards database, and relevance scores of genes ranking top 60 were used for screening Supplementary Material S6.

### Somatic mutation analysis

According to the somatic mutation data from the TCGA, we analyzed the data for each COAD patient using VarScan platform (Koboldt et al., 2012) and “maftools” R package. Our next step was to calculate the tumor mutation burden (TMB) of each patient and to compare the correlation between TMB and NRLPS risk groups. Additionally, a survival analysis was conducted based on the TMB score. Aside from that, the cBioPortal database was used to display somatic mutations of the selected genes within NRLPS.

### Differences in the effectiveness of chemotherapeutics and corresponding small molecule drugs

Based on the database called Genomics of Drug Sensitivity in Cancer (GDSC; https://www.cancerrxgene.org/) database, chemotherapy response for COAD patients could be predicted. A drug’s IC50 is determined by the dose, that is, required to result in 50% inhibition of cancer cells. To calculate the IC50 of drugs, the R package “pRRophetic” was used, followed by Wilcoxon signed rank comparisons of different IC50 values between high-risk and low-risk groups of NRLPS. A cutoff value of *p* < 0.05 was determined significant. The 3D structures of these drugs were obtained from the PubChem database.

### Statistical analysis

Statistical analysis was performed using the R software (version 3.6.3), Perl software (version 5.30 https://strawberryperl.com/). To determine related genes and their prognostic value, univariate Cox regression analysis was performed. The Kaplan-Meier method was utilized to generate the survival curves, and the log-rank test was used for comparison. We compared the results between groups using log-rank tests. Based on Spearman’s correlation analysis, the association between the prognostic signature and immune score was identified. m7G regulator expression levels were compared between COAD tissues and normal tissues using one-way analysis of variance. For each statistical analysis, a *p* < 0.05 represents a statistically significant difference.

## Results

### Identification of NRGs and their genetic variation landscape analysis

A flow diagram depicting the main steps of this study can be found in Graphical Abstract 1.

**FIGURE 1:**
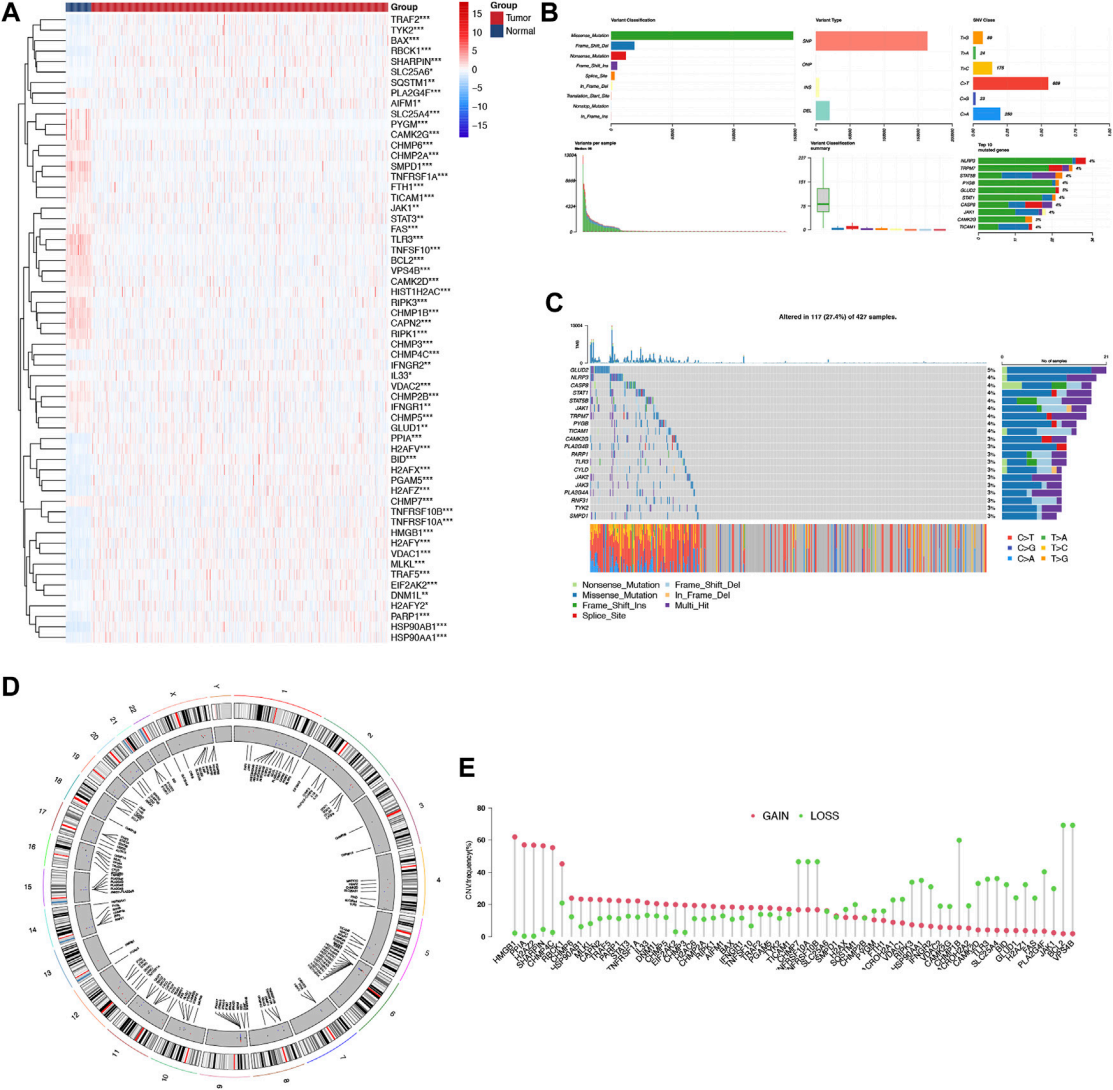
Landscape of genetic and expression variation of NRGs in COAD. **(A)** The differential expression of NRGs in COAD and normal colon tissues, Tumour, red; Normal, blue. **(B, C)** The mutation frequency and classification of NRGs in COAD. **(D)** The location of CNV alteration of NRGs on 23 chromosomes in the COAD cohort. **(E)** The CNV variation frequency of differentially expressed NRGs in the COAD cohort. The height of the column represented the alteration frequency.*p < 0.05,**p < 0.01,***p < 0.001, NRG necroptosis-related gene, COAD colon adenocarcinoma, SNP single nucleotide polymorphism, INS insertion, DEL deletion.

**FIGURE 2:**
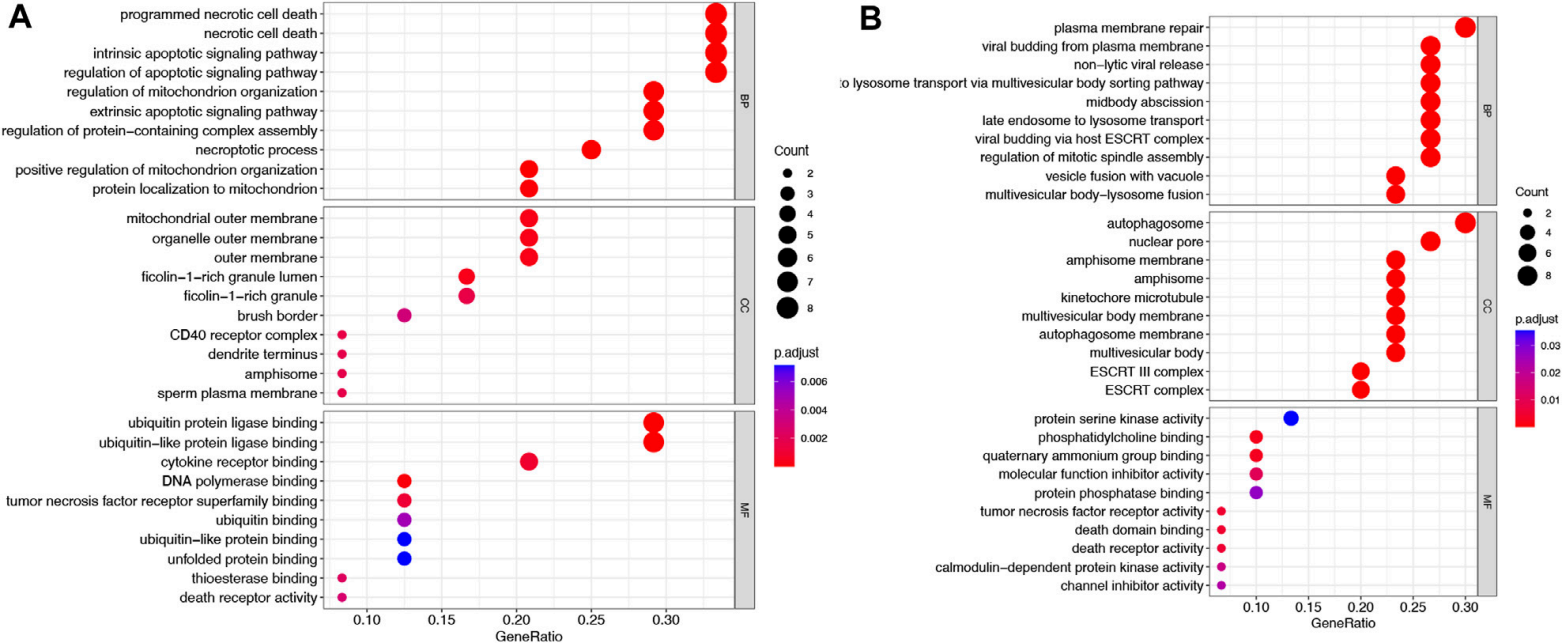
GO analysis of NRGs in COAD. **(A)** Upregulated NRGs **(B)** Downregulated NRGs.

Using the TCGA-COAD dataset, we first examined whether the NRGs are differentially expressed between COAD and normal colon tissues to explore their potential significance in carcinogenesis (FDR <0.05 and |logFC| >2). Figure 1A shows the identification of 60 Differential Expressed Genes (DEGs) (29 upregulated and 31 downregulated). To be specific, the expression of HSP90AB1, TNFRSF10B, PGAM5, H2AFY, PPIA, MLKL, H2AFZ, BID, TNFRSF10A, RBCK1, TRAF5, H2AFX, TRAF2, HSP90AA1, HMGB1, PARP1, VDAC1, CHMP4C, BAX, SHARPIN, TYK2, EIF2AK2, H2AFV, DNM1L, SQSTM1, H2AFY2, AIFM1, SLC25A6, and IL33 were increased, while the expression of TLR3, SMPD1, TNFSF10, CAPN2, CHMP1B, BCL2, VPS4B, TNFRSF1A, RIPK3, PYGM, RIPK1, CHMP6, CAMK2G, FAS, CAMK2D, FTH1, SLC25A4, CHMP3, CHMP2B, CHMP2A, PLA2G4F, VDAC2, TICAM1, HIST1H2AC, CHMP7, CHMP5, JAK1, IFNGR2, GLUD1, STAT3, and IFNGR1 were decreased comparing COAD with normal tissues. Following that, a comprehensive summary of CNVs and somatic mutations of the NRGs in COAD was then provided. A genetic mutation was detected in 117 of 427 (27.4%) COAD samples (Figures 1B, C), As far as variant classifications are concerned, missense mutations topped the list, while Single Nucleotide Polymorphisms (SNPs) represented the most common types. C > T was rated as the most common Single Nucleotide Variants (SNV) classification. The most frequently mutated gene, among the NRGs, was NLRP3, followed by TRPM7, STAT5B and PYGB (Figure 1C). A representation of the location of CNV alterations on chromosomes of these NRGs can be found in Figure 1D. Furthermore, differentially expressed NRGs had prominent CNV alterations, as revealed by Figure 1E. The next step is to explore the mechanisms and pathways by which NRGs are involved in the emergence and development of COAD. We conducted GO analysis of upregulated NRGs and downregulated NRGs separately (Figures 2A, B). It is important to note that these contents have important implications for the mechanism research of differential expression of NRGs in COAD.

### Identification of NRLs with significant prognostic value in COAD

Previous studies have demonstrated the importance of NRGs in the pathogenesis of COAD, but we also would like to investigate the potential value of NRLs in COAD.

**FIGURE 3:**
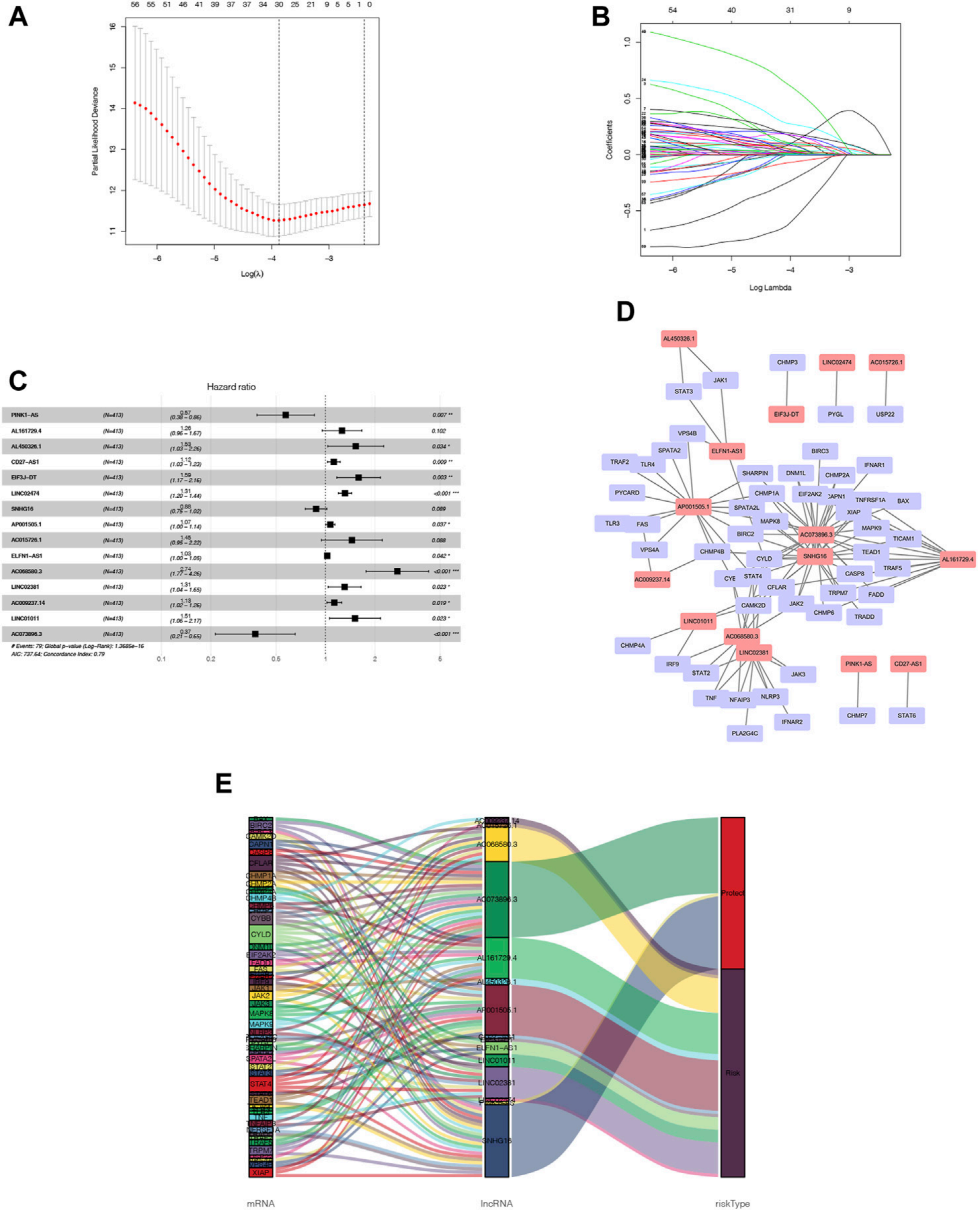
NRLPS development **(A, B)** LASSO etas performed to identify NRLs associated with COAD prognosis. **(C)** Signature construction by fifteen NRLs detect-ed using mullivariable Cox reptssion analysis. **(D, E)** The op-expression network between NRLs and NRGs in COAD visualized using Cytoscape software and Sankey diagram.

As a starting point, we retrieved 13,413 lncRNAs from TCGA-COAD cohort. Afterwards, with a threshold of *p*-value <0.001 and correlation coefficient |R| > 0.3 by the Pearson correlation analysis, we identified 1127 NRLs. A preliminary screening was also conducted using a univariate cox analysis, which identified 61 NRLs which were related to OS (*p* < 0.05), among which, 30 NRLs were subsequently screened out with LASSO regression analysis that was tenfold cross-validated (Figures 3A, B). We therefore conducted a multivariate Cox regression analysis and developed an NRLPS that contains 15 NRLs that can be used together to predict a patient’s outcome (Figure 3C). The forest plots revealed that all lncRNAs showed a considerable association with risk (HR > 1) with the exception of PINK1-AS and AC073895.3 (HR < 1). Besides, a co-expression network of lncRNAs and mRNAs was also visualized using Cytoscape software (Figure 3D). To illustrate whether these NRLs are protective or risk factors, we also drew a Sankey diagram using the R package “ggalluvial” (Figure 3E). Among these, LncRNA AC073896.3 had co-expression relationship with 24 NRGs (BAX, BIRC2, CAPN1, CASP8, CFLAR, CHMP1A, CHMP2A, CHMP6, CYBB, CYLD, DNM1L, EIF2AK2, FADD, IFNAR1, JAK2, MAPK8, MAPK9, SHARPIN, SPATA2L, STAT4, TRAF5, TRPM7 XIAP and TEAD1), SNHG16 was co-expressed with 23 necroptosis-related genes (BIRC2, BIRC3, CAMK2D, CAPN1, CASP8, CFLAR, CHMP1A, CHMP2A, CHMP4B, CYLD, DNM1L, EIF2AK2, JAK2, MAPK8, MAPK9, SPATA2L, STAT4, TICAM1, TNFRSF1A, TRADD, TRPM7, XIAP, and TEAD1) and so on.

### Verifications of NRLs expression in clinical COAD samples and cell lines

**FIGURE 4:**
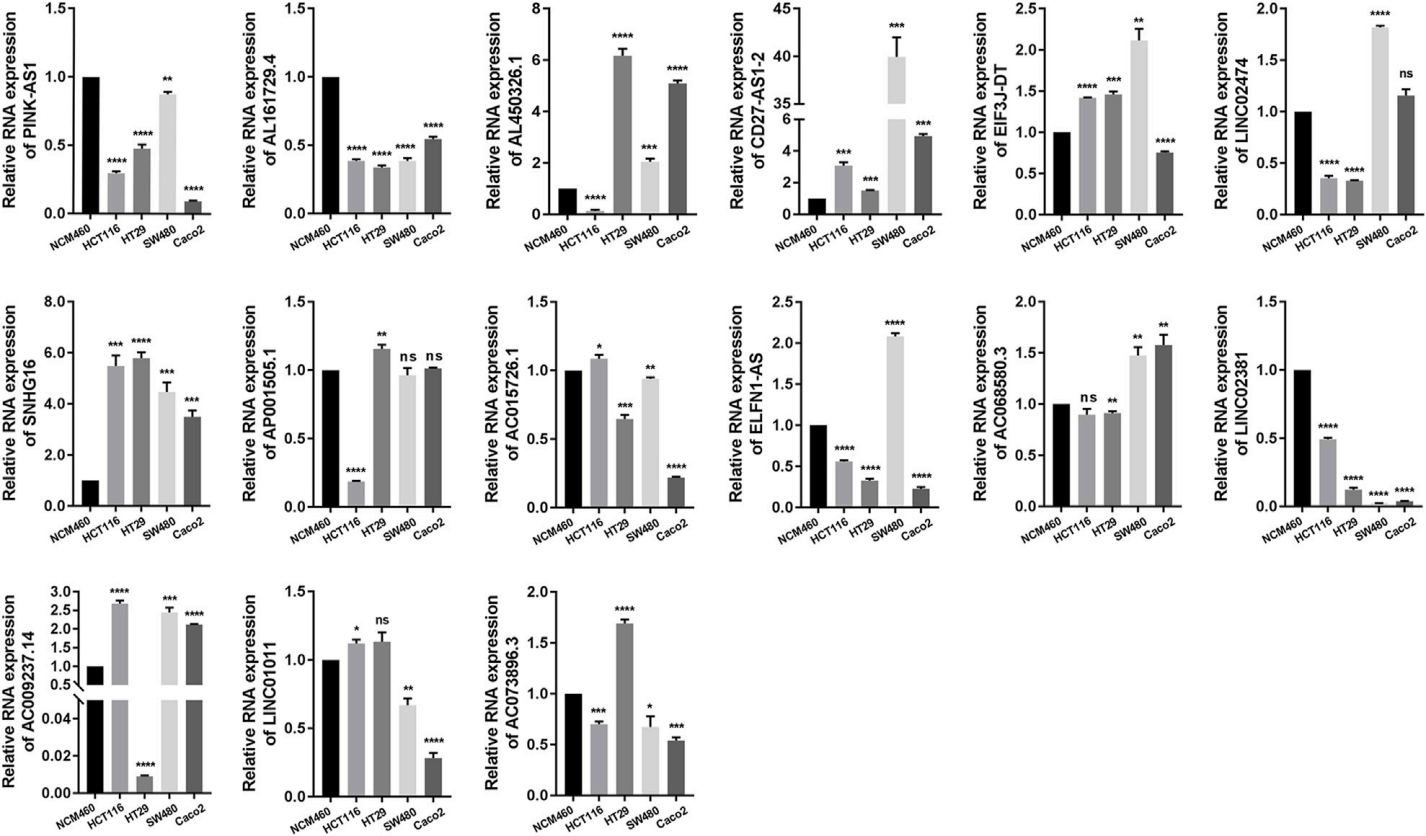
qRT-PCR resluts of 15 NRLs expression in different colon cancer cell lines.

The expression of each NRL in the NRLPS was analyzed in COAD, and the following step was to validate our findings by using qRT-PCR in COAD tissues and cell lines (Figure 4 and Supplementary Figure S1). Results showed that CD27-AS1, SNHG16, ELFN1-AS1, LINC01011, LINC02474, SNHG16, AP001505.1, and AC068580.3 expressions were significantly higher, while PINK-AS1, LINC02381, AC015726.1, and AL450326.1 were significantly lower in the COAD tissues than in the normal tissues. Moreover, for each lncRNA, at least one COAD cell line further supported this conclusion. Interestingly, the expression level of CD27-AS1-2, PINK-AS1, SNHG16, and LINC0238 showed completely the same tendency in tissue samples and different cell lines. However, exceptions existed. AC073896.3 was lowly expressed in COAD tissues which was contrary to the cell lines expression.

### Prognostic value of NRLPS risk score

**FIGURE 5:**
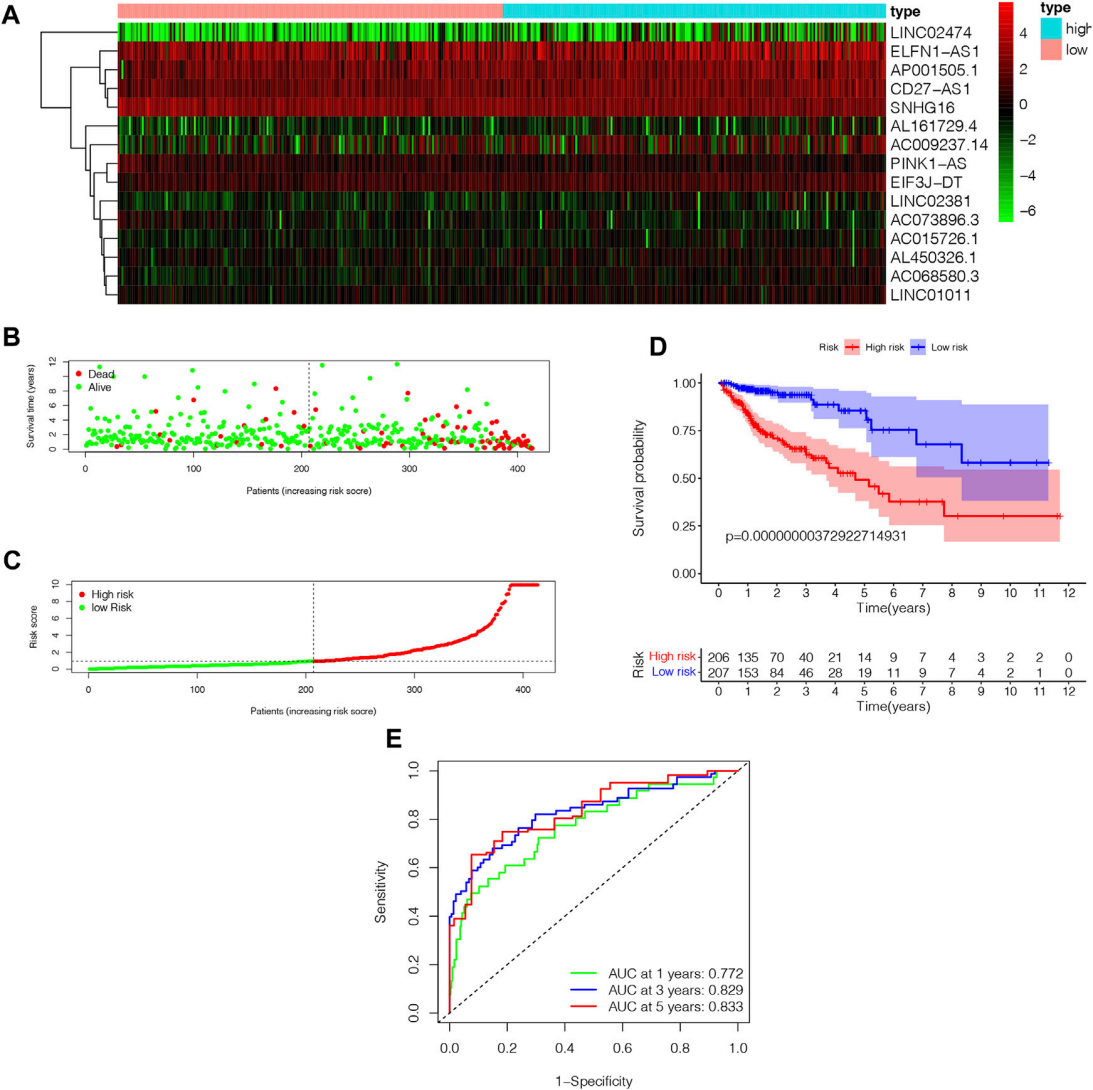
The analysis of NRLPS fix patients with WAD. **(A)** Hcatmap of NRI-s˙ expression in risk subgroup. **(B)** The survival time of the patients. **(C)** The risk score between two risk gmups. **(D)** The KM survival curve of NMI'S risk store. **(E)** The art. ds under the ROC curve about I-y = 0-year and 5-year.

In order to determine the high-risk and low-risk groups of patients with COAD, we divided them according to their median risk score. In Figure 5A, the heatmap illustrates how 15 NRLs show different expressions depending on the subgroups. People with high risk scores had a higher mortality rate and a worse prognosis, according to a distribution of risk scores and survival status (Figure 5B, C). As a result of evaluating Kaplan-Meier survival curves, the prognoses of different risk subgroups were also evaluated; those in high-risk groups had a shorter OS than those in low-risk groups, as shown in Figure 5D (*p* < 0.001). We generated ROC curves for 1-year, 3-year, and 5-year for our risk signatures and calculated AUC values, which are 0.772, 0.829, and 0.833, respectively (Figure 5E), demonstrating our score’s reliability and sensitivity over the long term.

### NRLPS verification and nomogram construction

**FIGURE 6:**
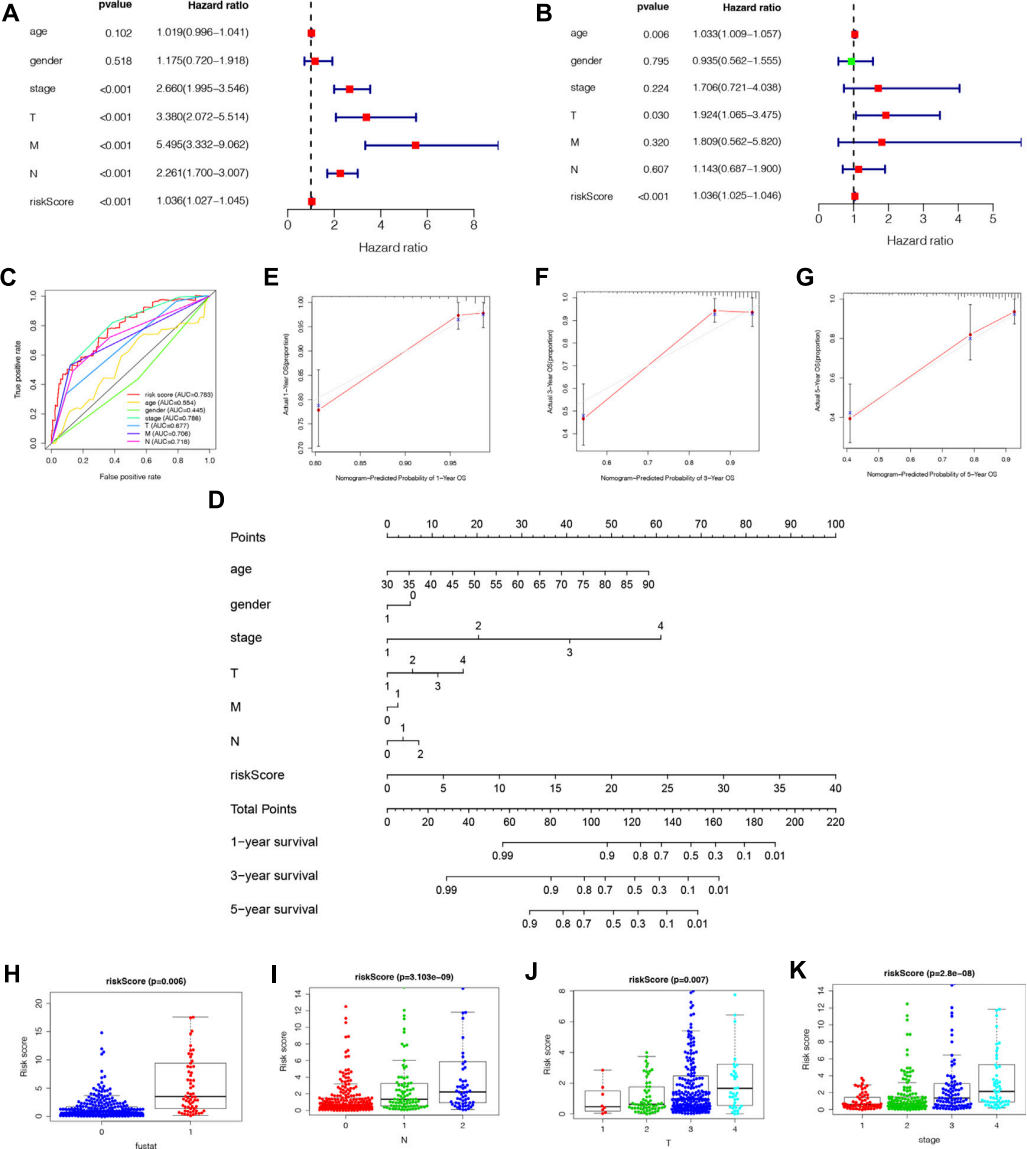
**(A)** Univariate cox regression analysis identifies factors related to patient survival. **(B)** Multivariate cox regression analy-sis identifies independent prognostic factors. **(C)** Multi-ROC curves of NRLPS risk score and clinical traits. **(D)** A nomogram was developed to predict 1-, 3-, and 5-year survival. Calibration curves showing nomogram predictions for 1-year **(E)**, 3-year **(F)**, and 5-year **(G)** survival. NRLPS was associated with the clinical features of patients with COAD: survival outcome [**(H)**, *p* < 0.001]; (N) [**(I)**, *p* < 0.001)]; (T) [**(J)**, *p* < 0.001]; K stage [**(K)**, *p* < 0.001]. T, tumor size; N, regional lymph node metastasis.

We performed univariate and multivariate Cox regression analyses to determine if NRLPS was an independent predictor of survival. A univariate cox regression analysis revealed the following results: HR = 1.036 and 95% CI: 1.027–1.045 (*p* < 0.001). The results of the multivariate Cox regression analysis were as follows: HR = 1.036 and 95% CI: 1.025–1.046 (*p* < 0.001), suggesting that the NRLPS risk score plays an important role in determining prognosis, regardless of age, gender and TNM stage (Figures 6A, B). Additionally, we examined whether the risk score has a better prognostic significance compared to other clinicopathological factors shown in Figure 6C. We also developed a nomogram based on the risk score for possible clinical use in predicting the prognosis of patients (Figure 6D). Further, the calibration curves in Figures 6E–G were used to validate the accuracy of the nomogram model by comparing predictions with actual survival rates of COAD patients. Overall, these results demonstrated that our risk signature was capable of indicating a high level of reliability and sensitivity.

### NRLPS and clinical features of COAD patients

**FIGURE 7:**
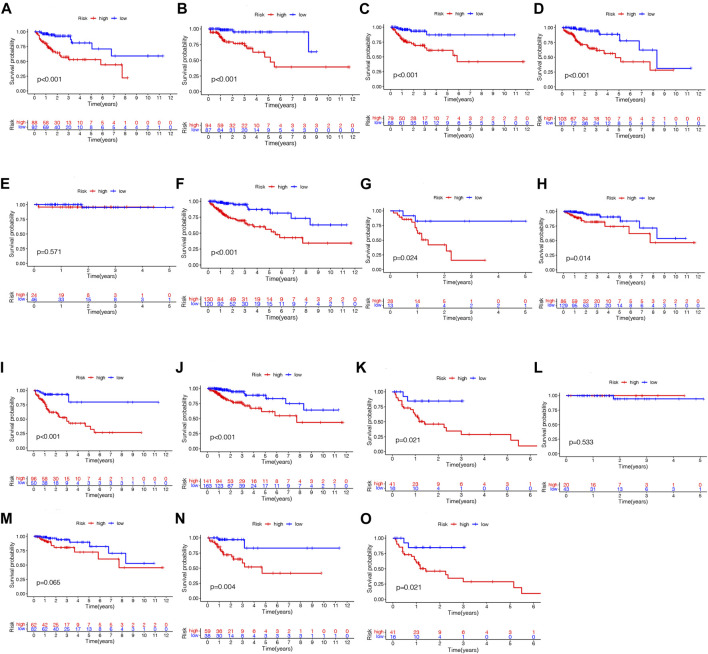
Kaplan-Meier plots depicting subgroup survival predicted by NRLPS risk score. Patients aged >68 years **(A)** and 568 years **(B)**; Female **(C)** and male **(D)** patients; T1-2 **(E)**, T3 **(F)**, T4 **(G)**; NO **(H)** and N1-2 **(I)**; MO **(J)** and MI **(K)**; Stage I **(L)**, II **(M)**, III **(N)** and IV **(O)**. PS fix patients with WAD. **(A)** Hcatmap of NRI-s˙ expression in risk subgroup. **(B)** The survival time of the patients. **(C)** The risk score between two risk gmups. **(D)** The KM survival curve of NMI'S risk store. **(E)** The art. ds under the ROC curve about I-y = 0-year and 5-year.

As a further assessment of NRLPS’ role in development of COAD, we correlated it with clinicopathological factors. A high-risk score reveals a significantly worse prognosis when compared with a low-risk score (Figure 6H, *p* < 0.001). As shown in Figures 6I–K, there were significant correlations between the risk score and tumor stage (*p* < 0.001), tumor size (*p* < 0.001) and lymph node metastasis (*p* < 0.001). In Figure 7, survival analysis of clinical stratification analysis in regard to our signature was performed, including age, gender, grade and TNM stage. These results indicate that NRLPS risk score is closely associated with COAD progression and may serve as a reliable tool for predicting COAD survival.

### Differences in NRLPS between high-risk and low-risk groups as well as functional enrichment analysis

**FIGURE 8:**
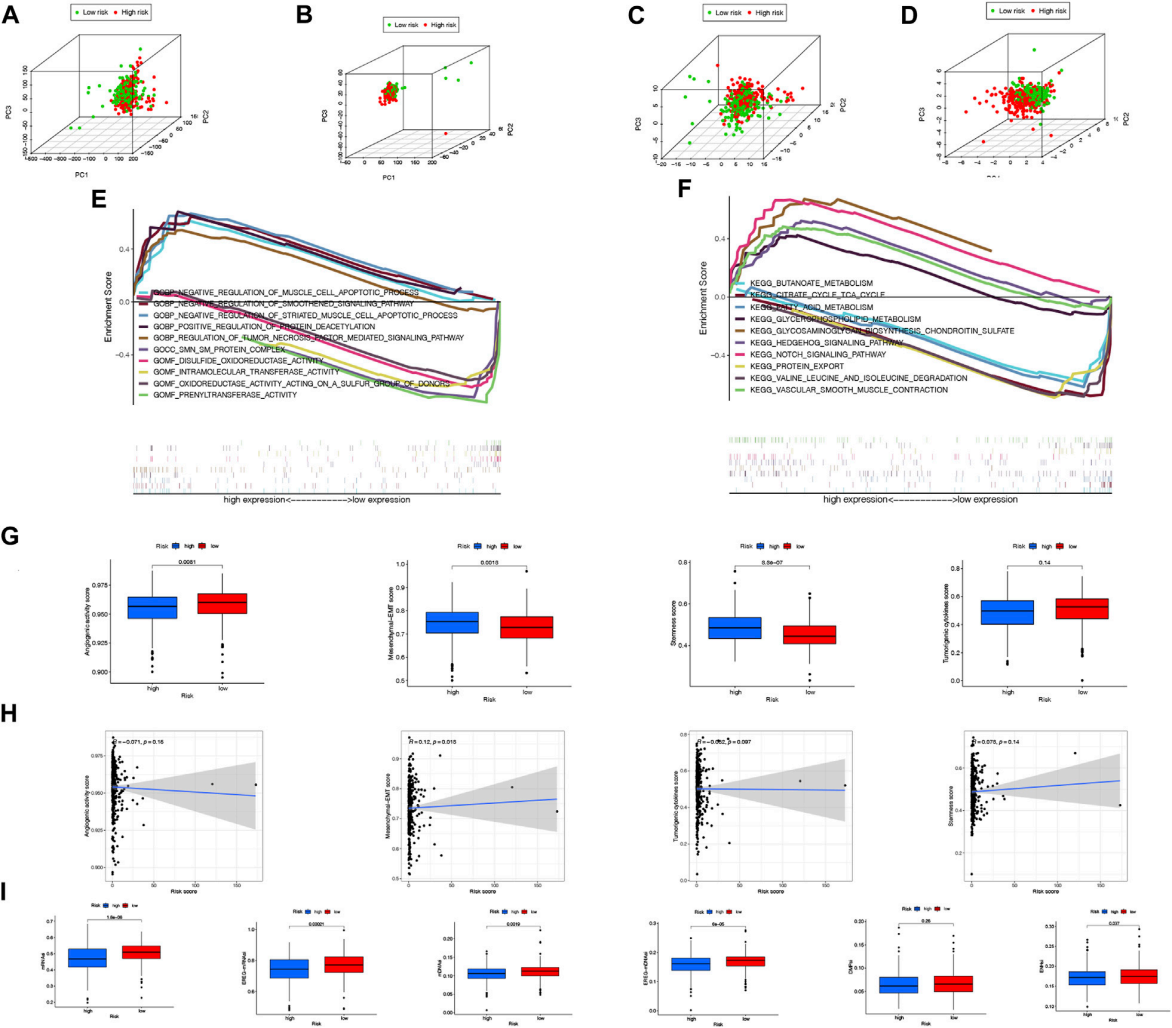
PCA illustrations based on the whole-genome **(A)**, all lncRNAs **(B)**, NRLs **(C)** and NRLPS risk score **(D)**. GSEA results showing differen-tial enrichment of genes in GO **(E)** and KEGG **(F)** with NRLPS risk score. **(G)** Differences of angiogenic activity, mesenchymal-EMT, tumourigenic cytokines and sternness scores between the high- and low-risk groups. **(H)** The correlation of the risk score and angiogenic activity, mesenchy-mal-EMT, tumourigenic cytokines and sternness scores. **(I)** Differences of TSIs between the two groups. (*p < 0.05; **p < 0.01; ***p < 0.001; ns, not significant).

In Figures 8A–D, PCA was used to determine whether there is a difference in necroptosis distribution across the genome-wide expression profile, lncRNAs, NRGs, and NRLPS. In comparison to the other 3 methods, NRLPS allowed for a more obvious division of patients into low- and high-risk groups.

The differentially expressed genes between high-risk and low-risk groups were analyzed to determine the physiological functions and signal transduction pathways associated with the NRLPS in COAD. The majority of enriched GO terms in Figure 8E were related to “_NEGATIVE_REGULATION_OF_SMOOTHENED_SIGNALING_PATHWAY”, “POSITIVE_REGULATION_OF_PROTEIN_DEACETYLATION”,“REGULATION_OF_TUMOR_NECROSIS_FACTOR_MEDIATED_SIGNALING_PATHWAY”,“INTRAMOLECULAR_TRANSFERASE_ACTIVITY” and “PRENYLTRANSFERASE_ACTIVITY” (Figure 7A). In addition, the analysis of KEGG pathways in Figure 8F was mainly focused on“BUTANOATE_METABOLISM”, “CITRATE_CYCLE_TCA_CYCLE”,“GLYCEROPHOSPHOLIPID_METABOLISM”,“HEDGEHOG_SIGNALING_PATHWAY” and “NOTCH_SIGNALING_PATHWAY” (Figure 7B).

In addition, we sought to determine whether tumor development is associated with angiogenic activity, EMT, tumorigenic cytokines, and stemness scores in COAD patients. In Figure 8G, it is shown that the high-risk group had higher EMT and stemness scores. In Figure 8H, the correlation between the risk score and four indices is shown with R = 0.12 and *p* = 0.018 suggesting a positive association between the risk score and the mesenchymal EMT score. In addition, the high-risk group had lower TSIs, such as mRNAsi, EREG-mRNAsi, mDNAsi, EREG-mDNAsi and ENHsi (Figure 8I). Equally important, we found our risk score showed a significant correlation with oxidative stress genes in Supplementary Figure S2.

### Immunity and gene expression

**FIGURE 9:**
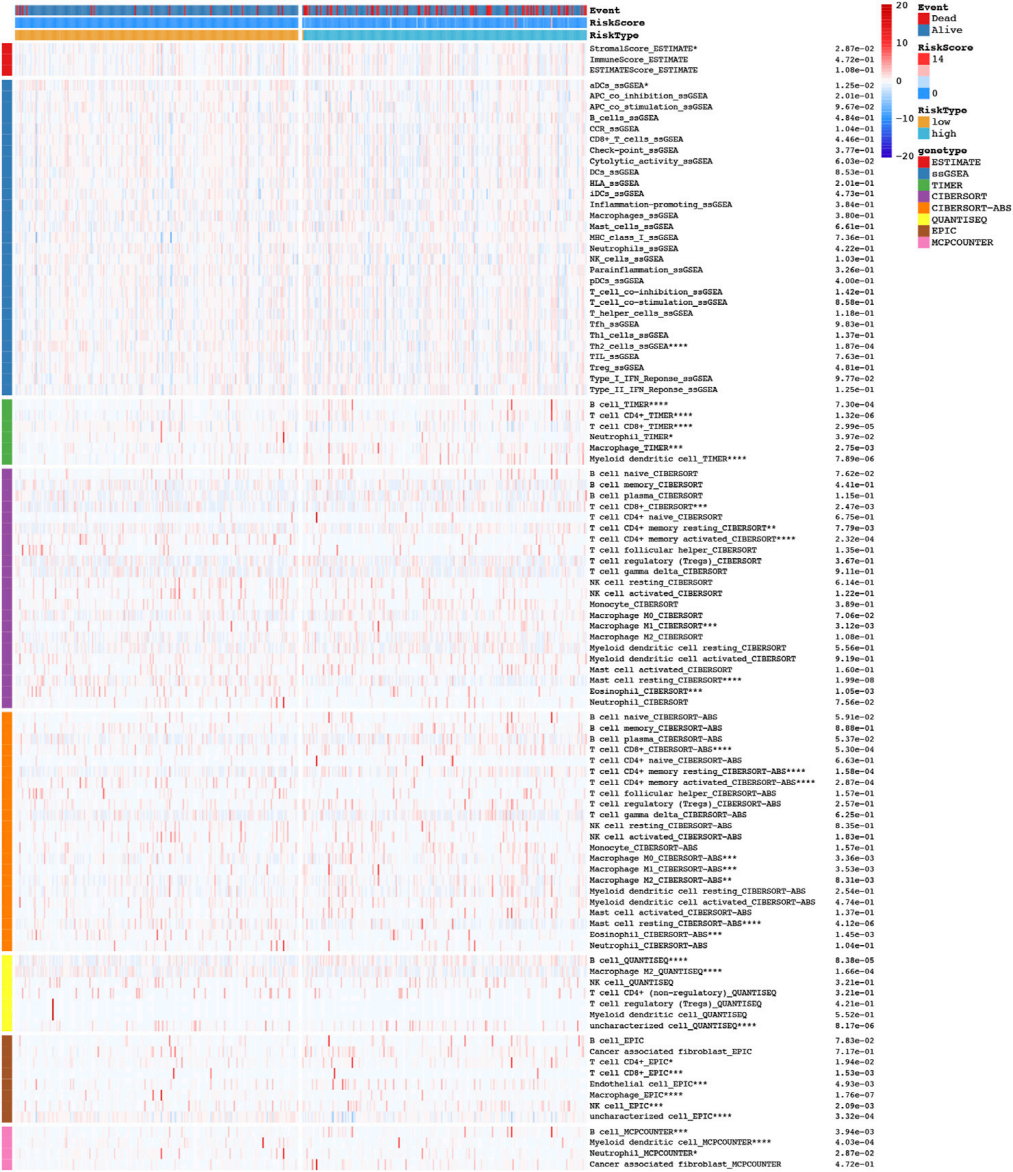
The heatmap of immune landscape among high risk and low risk groups based on NRLPS by using the CIBERSORT, CIBERSORT-ABS, ESTIMATE, MCPcounter, ssGSEA, QUANTISEQ, EPIC and TIMER algorithms. Adjusted *p* values were showed as: *p < 0.05; **p < 0.01; ***p < 0.001.

The tumor microenvironment has previously been implicated in tumor development (Hanahan and Weinberg, 2011). As shown in the heatmap of in Figure 9, we further explored the immune landscape of COAD patients using eight types of algorithms to further understand the relationship between NRLs and tumor immunity. A TIMER database, for example, showed significant correlations between immune cells in different risk groups, including B cells, CD4^+^ T cells, CD8^+^ T cells, neutrophils, macrophages, and dendritic cells.

**FIGURE 10:**
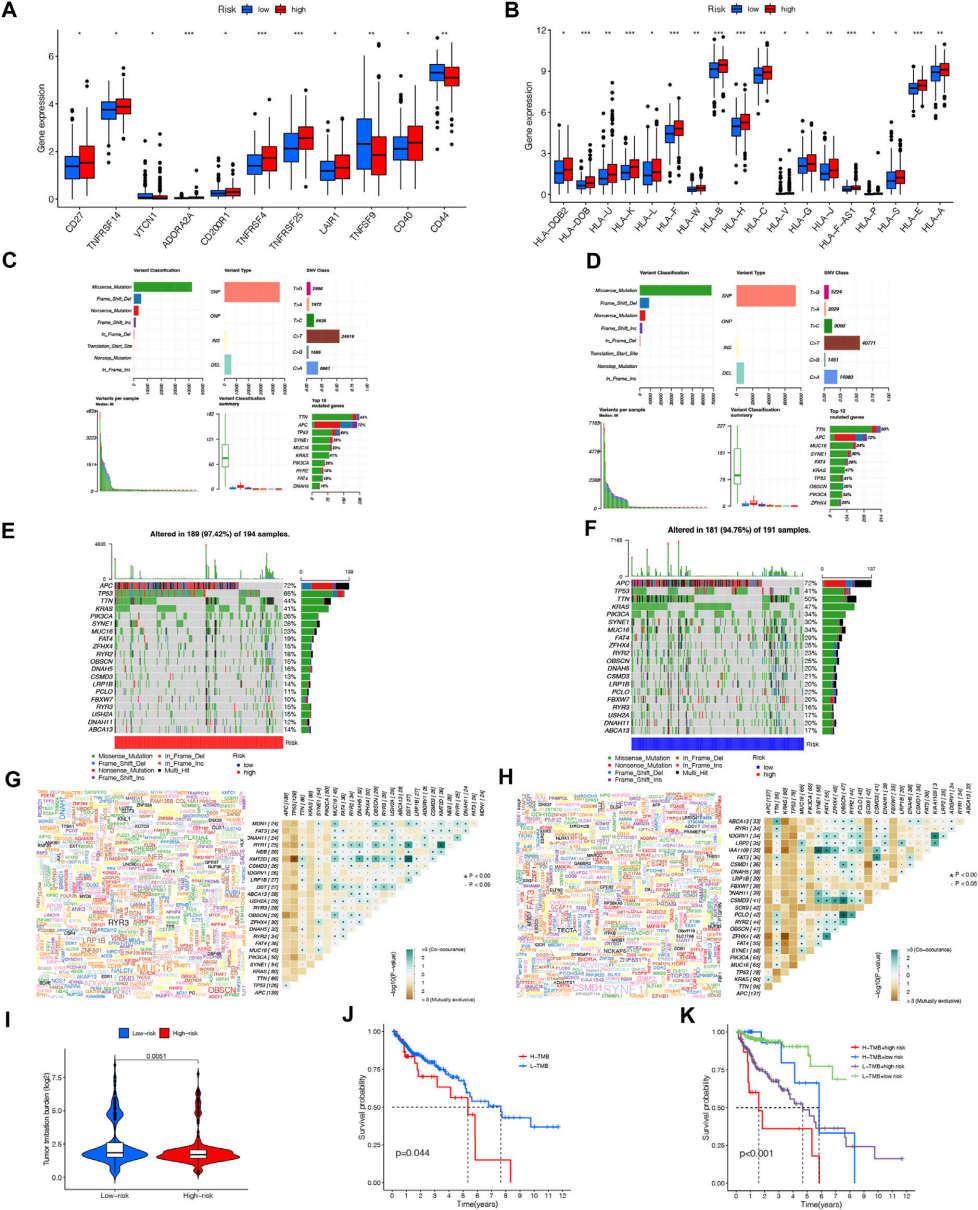
Immune checkpoint genes expression level **(A)**, MHC molecules expression level **(B)** between the high- and low-risk groups. (*p < 0.05; “*p* < 0.01; ***p < 0.001; ns, not significant). Distribution of mutation types between the high-risk **(C)** and low-risk groups **(D)**. Waterfall maps of the somatic mutations in the high-risk group **(E)** and the low-risk group **(F)**. Genecloud and heatmap of co-occurrence and mutually exclusive mutations of the differently mutated genes in the high-risk group **(G)** and the low-risk group **(H)**. sp < 0.01. **(I)** Comparison of TMB between the high- and low-risk groups. **(J)** Difference in overall survival between high TMB and low TMB groups. **(K)** Difference in overall survival based on TMB and risk score.

TNFRSF14, TNFRSF4, TNFRSF25, LAIR1 CD40 and CD200R1 were significantly higher expressed in the high-risk group than in the low-risk group using boxplots to visualize differentially expressed ICGs in different risk subgroups (*p* < 0.001). The remaining ICIs such asVTCN1, ADORA2A, TNFRSF9, CD44, and CD27 also had statistically significant differences (Figure 10A). In addition, MHC molecules were mostly detected in the high-risk group and their expression was significantly increased (Figure 10B). The results of these studies showed that the risk model was reliable for predicting how COAD patients would respond to corresponding ICIs.

### Comparison of somatic mutation and TMB in NRLPS

We downloaded nucleotide variation data from TCGA in order to see if there are any differences in mutations between high-risk and low-risk groups. As shown in Figures 10C, D, the information concerning mutated genes is presented in terms of the variant classification, variant type, and single nucleotide variant (SNV) class. It is shown in the waterfall plot that the top twenty genes with the highest mutation frequency in high- and low-risk groups are represented by mutational landscape in Figure 10C and Figure 10D. APC (72%), TP53 (65%), TTN (44%), KRAS (41%), and PIK3CA (26%), were the most common mutated genes in the 189 samples (97.42%) of the high-risk group, while APC (72%), TP53 (41%), TTN (50%), KRAS (47%) and PIK3CA (34%) were the top 5 mutations in the low-risk group (Figure 10E; Figure 10F).

There was also evidence of somatic mutation interactions. Most genes showed cooccurrence of mutations, and TP53-KMT2D and TP53-ZFHX4 mutations were found in the high-risk and low-risk group to be mutually exclusive, respctively (Figure 10G; Figure 10H). In addition to comparing TMB between the two groups, significant differences were found in survival time (*p* = 0.0044) between the groups with high-TMB and low-TMB. The prognosis of patients in the high-risk group with a high TMB was significantly worse compared to those in the low-risk group with a low TMB when combined with our NRLPS model (Figures 10I–K).

### Chemosensitivity determined in COAD patients using risk scores

As of now, chemotherapy drugs continue to be the primary treatment for COAD. Chemoresistance, however, is a major factor contributing to COAD patients’ poor prognoses.

**FIGURE 11:**
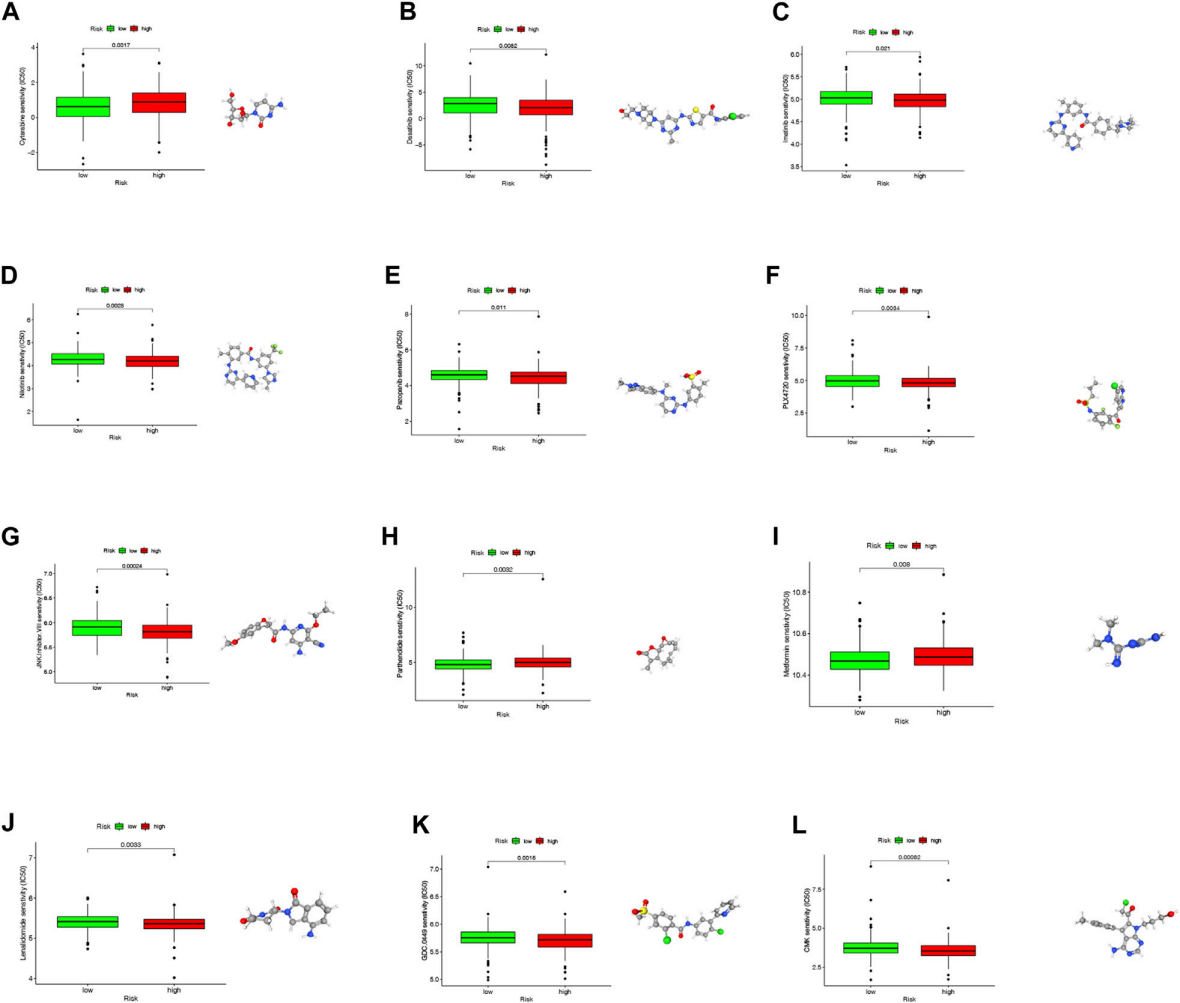
**(A–L)** The differences in the chemotherapy response of common chemotherapy drugs between the high- and low-risk groups, along with their 3D structure screened out from the Pubchem database (*p < 0.05; **p < 0.01; ***p < 0.001; NS, not sig-nificant).

We investigated the sensitivity of COAD patients to common chemotherapy drugs using GDSC project in both high- and low-risk groups (*p* = 0.0017 for Cytarabine, *p* = 0.0082 for Dasatinib, *p* = 0.021 for Imatinib, *p* = 0.0028 for Nilotinib, *p* = 0.011 for Pazopanib, *p* = 0.0034 for PLX4720, *p* = 0.00024 for JNK. Inhibitor.VIII, *p* = 0.0032 for Parthenolide, *p* = 0.008 for Metformin, *p* = 0.0033 for Lenalidomide, *p* = 0.015 for GDC.0449, *p* = 0.00082 for CMK). The low-risk group exhibited increased IC50 values for Dasatinib, Imatinib, Nilotinib, Pazopanib, PLX4720, Lenalidomide, GDC.0449 and CMK, indicating that high-risk patients may benefit from these chemotherapy agents. Interestingly, the low-risk group exhibited decreased IC50 values for Cytarabine, JNK. Inhibitor.VIII, Parthenolide and Metformin, indicating that low-risk patients may benefit from these chemotherapy agents. Overall, these results showed that NRLPS was related to drug sensitivity. The 3D structures of these drugs were displayed through the PubChem database (Figures 11A–L).

## Discussion

It is estimated that COAD is the most common type of colorectal cancer in China, and its incidence is steadily increasing each year, resulting in increased economic and social costs (Shi et al., 2021). And as a result of COAD’s adverse prognosis, it is crucial that we uncover new, better and more accurate predictive markers as well as improve the prognosis (Taieb et al., 2019). A number of studies have shown that the onset of necroptosis induces antitumor immunity and has a significant influence on tumor progression and metastasis (Gong et al., 2019). Likewise, lncRNAs have been implicated in COAD metastasis, EMT, formation of stemness and chemoresistance (Wang et al., 2019). The roles of COAD’s NRLs as potential biomarkers and therapeutic targets, however, have not been extensively studied.

In our study, the first step was to identify NRGs based on the KEGG database and perform a differential expression analysis of COAD samples in comparison to normal samples, demonstrating that 60 NRGs showed significant differences in expression levels between the two groups (upregulation of 29 NRGs; downregulation of 31 NRGs). Afterwards, we described the expression landscape of NRGs in COAD by documenting genetic mutations of NRGs and the localizations of NRGs CNV alterations. In light of the importance of lncRNAs in COAD, 1127 lncRNAs related to necroptosis were obtained for our follow-up study. Hence, we identified 15 NRLs with prognostic value for COAD through univariate COX regression, LASSO analysis, and multivariate COX regression analysis, based on which our risk model was constructed. Furthermore, we validated the expression of these lncRNAs in multiple cell lines and tissue samples. Reviewing the literature, we discovered that some of these 15 NRLs that construct our risk models have also been reported elsewhere. For example, in a signature of ferroptosis-related lncRNAs, LINC02381, ELFN1-AS1, LINC01011, AL450326.1, and AL161729.4 contributed to COAD carcinogenesis and correlated closely with ICGs expression (Chen et al., 2022). A study by Du et al. (2020) found that ELFN1-AS levels are significantly elevated in COAD, which increases invasiveness and prevents tumor cells from apoptosis by affecting miR-191-5p/SATB1 axis. Also, ELFN1-AS1 partially suppresses MEIS1 in CRC by suppressing epigenetic activity of EZH2-DNMT3a, which promotes resistance to chemotherapy (Li et al., 2022). As Christensen et al. reported, there was an upregulation of the SNHG16 gene expression in CRCs which was mediated by Wnt signaling (Christensen et al., 2016). As an activator of the Hedgehog pathway via miR-802/PTCH1, SNHG16 is also upregulated in cancer stem cells (CSCs) (Zhang et al., 2022). And SNHG16 can boost CRC epithelial-mesenchymal transition (EMT) and promote liver metastasis through YAP1 (Xiang et al., 2022). It was discovered by Du et al. (2021) that LINC02474 inhibited CTLs and NKs expression of granzyme B in CRC tumor microenvironment, promoting tumor invasion and metastasis. Our findings further support the idea that our selected lncRNAs play a significant role in the development of COAD, which strengthens the credibility of the predictions and mechanistic explorations we make in this study.

A median risk score of COAD patients was subsequently calculated, and patients were then classified into high-risk and low-risk groups. There were significant differences between the groups according to the Kaplan-Meier curves, with the high-risk group having a worse survival prognosis and COAD patients having short survival times and more deaths with increasing risk scores. Additionally, its AUC values increased over time at 1, 3, and 5 years. Taking the clinical characteristics into account, the total AUC value was 0.783, which is comparable to the TNM stage. In contrast, COX multivariate analysis did not show an independent prognostic significance for TNM stage with a *p*-value greater than 0.05. To further assess the prognosis of patients, we drew an additional nomogram of NRLPS and clinicopathological characteristics. Interestingly, we found that the predictive plots for 1-, 3-, and 5-year survival were closer to the true curve, showing the potential clinical value of our risk score on a clinical level. The risk score also displayed high predictive capability in assessing prognosis for clinicopathological subgroups based on clinical characteristics such as patient age, gender, and TNM stage. Overall, the risk score signature based on 15 NRLs has proven to be a reliable and highly useful prognostic factor for COAD, and has displayed excellent performance, which is of clinical significance.

We then analyzed necroptosis-related signaling pathways within high- and low-risk COAD groups by applying GSEA, and then filtered through the five pathways with the highest upregulations and lowest downregulations. Consistent with our analysis, Notch signaling pathway expression was upregulated in COAD. There is evidence that Notch signaling is involved in regulating heterotypic interactions between stroma and tumors in the tumor microenvironment. Multiple aspects of tumor biology have been demonstrated to be mediated by this signaling pathway, including angiogenesis, the maintenance of CSCs, immune infiltration, and the response to chemotherapy (Meurette and Mehlen, 2018; Jackstadt et al., 2019). It has been discovered that epithelial NOTCH1 signaling is a prognosis-damaging subtype of CRC, and that it is capable of driving tumor cell metastasis through TGF β-dependent migration of neutrophils (Jackstadt et al., 2019). Further, our results indicate that Hedgehog and Smoothened (Smo) signaling pathways are upregulated in COAD, which has been regarded as a positive regulator of the Wnt signaling pathway, important for the survival of colon CSCs (Regan et al., 2017). COAD progression has been inhibited in some instances by targeting signaling pathways involved in CSC regeneration and differentiation, including Notch, WNT, and Hedgehog (Ma et al., 2017).

Next, we examined the relationship between the risk score and stemness scores, EMT, tumor angiogenesis, which are believed to contribute to tumor progression and metastasis. Stemness scores and EMT have been found upregulated in high-risk group significantly. The proliferation of CSCs has been attributed to cancer metastasis, recurrence, and chemoresistance, and the presence of high CSC scores often indicates a poor prognosis for colon cancer (Patsalias and Kozovska, 2021). Besides, as mentioned above, NRL SNHG16 is believed to be relevant to the stemness of CRC. Moreover, NRGs are also associated with CRC stemness. For example, overexpression of RBCK1 in CRC cells enhances cell stemness and chemoresistance (Liu et al., 2019). HMGB1 is highly expressed in COAD tissues, and the overexpression of HMGB1 in glioblastoma is thought to promote self-renewal of glioma stem cells (Ye et al., 2022). While IL33 can dually target tumor cells and macrophages to promote stem cell production in colon cancer to drive tumor progression (Fang et al., 2017). EMT is a critical process for metastasis and progression in COAD, likewise, suggesting a very poor prognosis. However, tumor angiogenesis is not significantly different in the two groups. Additionally, tumor angiogenesis contributes to tumor metastasis and formation as well, which can lead to the development of CSCs as well (Catalano et al., 2013). Our hypothesis is that simple necrosis often occurs within solid tumors, since internal poor neovascularization and the deprivation of nutrients and oxygen (Yan et al., 2022). The high-risk group is more likely to experience tumor angiogenesis, which could lead to a poorer outcome. Unfortunately, there are no relevant studies that demonstrate the correlation between necroptosis and tumor angiogenesis, which needs to be investigated further in future studies.

It is generally believed that in malignant patients with high TMB, more antigens are induced that could enhance tumor immunogenicity, by which ICI response are more prevalent, leading to a better prognosis (Jardim et al., 2021). A recent study suggested that TMB may play a role in predicting the success of immunotherapy in some cases (McGrail et al., 2018). Accordingly, a possible synergistic role of necroptosis in patients with COAD can be posited where by necroptosis, a process that also increases tumor immunogenicity, acts in conjunction with TMB. Our study investigates TMB in both risk groups and discovers that TMB is lower in the high-risk group. The top five genes with the highest mutation frequency in the high-risk group were APC (72%), TP53 (65%), TTN (44%), KRAS (41%) and SYNE1 (28%). Of these, APC mutation is one of the earliest events in CRC initiation, and the major role of APC in CRC is thought to be related to its negative regulation of Wnt signaling pathway by targeting β-catenin degradation. CRC also shows high mutations of KRAS and TP53, which cooperate with APC mutation, to drive CRC progression and invasion (Caspi et al., 2021). Moreover, CRC patients with KRAS mutation often fail to respond to treatment with EGFR inhibitors (Parseghian et al., 2019). However, according to the results of the survival analysis, the overall survival of patients with high TMB is instead decreased, and the inclusion of the risk score further reveals that the survival of the high-risk group is still worse. In contrast, the survival rate of patients with high TMB was higher than those with low TMB in the early years, while the survival rate plummeted in the following years. In fact, there is still considerable disagreement on whether TMB can be a reliable biomarker (McGrail et al., 2021). High TMB could mediate drug resistance, T-cell dysfunction, chromosomal instability and genetic heterogeneity (Valero et al., 2021). When considering the context of the immunotherapy received by the patient, higher TMB is not associated with a better prognosis in certain cancer patients who received non-ICI therapy, but rather suggests a worse prognosis (Valero et al., 2021). Therefore, this difference in prognosis is perhaps due to the fact that patients included in the cohort have the absence of immunotherapy, and the development of drug resistance, distant metastases, and other comorbidities, which still need to be further investigated.

An TME is a regulated environment where tumor cells grow in the presence of non-tumor cells and other immune-related elements (Xiao and Yu, 2021). There is a growing understanding that necroptosis is an immunogenic cell death (ICD) process which enhances the immunogenicity of tumors (Aaes and Vandenabeele, 2021). When necroptosis is activated in the TME, immunostimulatory cytokines are produced, which facilitate the infiltration of immune cells that are responsible for the anti-cancer effect (Park et al., 2021). A comprehensive immune cell infiltration analysis of two risk groups was then conducted using ESTIMATE, XCELL, TIMER, CIBERSORT, CIBERSORT-ABS, QUANTISEQ, EPIC, and MCPcounter algorithms to explore COAD immune susceptibility from “cold” to “hot” therapeutic targets. According to TIMER results, significant differences were found in the levels of CD8^+^ T cells, dendritic cell DCs, CD4^+^ T cells, B cells, macrophages and neutrophils between the two risk groups. Study demonstrated that TME undergoing necroptosis with the release of DAMPs (Krysko et al., 2012) and activation of NF-κB signaling (Snyder et al., 2019) can recruit and activate DCs, which are essential for cross-initiation and infiltration of CD8^+^ effector T cells into the TME (Spranger et al., 2017), through the assistance of CD4^+^ helper T cells, thus triggering a sustained cytotoxic antitumor immune response leading to colon cancer cell death (Minute et al., 2020). Van et al. validated that induction of necroptosis by intra-tumoral delivery of mRNA encoding MLKL to colon cancer models inhibited tumor formation and distant metastasis, mechanistically through rapid triggering of tumor antigen-specific CD4 and CD8 T-cell responses, which required Batf3-dependent cDC1 and DC migration (Van Hoecke et al., 2018). A significant difference was also observed between the risk groups in terms of the expression of ICGs. In the high-risk group, the expression of TNFSF9 was remarkably reduced. As a co-stimulatory ligand for the 4-1BB receptor, TNFSF9 promoted CD4 T cell and CD8 T cell activation, proliferation, and survival and was highly expressed in DCs maturation (Chester et al., 2018). There is currently a strong correlation between the lack of anti-tumor T-cell infiltration in some COAD patients and the ineffectiveness of immune checkpoint inhibitors-based immunotherapy (Lizardo et al., 2020). Necroptosis, however, provides a potential therapeutic strategy by increasing the immunogenicity of COAD and improving the efficacy of T-cell therapies, offering a foundation for guiding individualized immunotherapy.

Patients with intermediate to advanced COAD are currently treated primarily with chemotherapy (Taieb et al., 2019). Nevertheless, since mechanisms such as EMT, CSC, and hypoxia can induce multiple drug resistance (MDR) (Shibue and Weinberg, 2017), the use of our risk score is essential for detecting patients who are sensitive to chemotherapy drugs. Patients who fall into the high-risk group are more sensitive to chemotherapy agents such as JNK. Inhibitor.VIII, Lenalidomide, GDC.0449, and CMK. COAD with low or absent PD-L1 expression were induced to become apoptotic and chemoresistance was inhibited with JNK inhibitors (Sun et al., 2021). As a result of GDC.0449 acting on Smo, colon cancer cells were able to grow slower and undergo less EMT as a result of reducing Hedgehog pathway activity (Magistri et al., 2017). A major benefit of lentidomide is that it may normalize tumor vessels and improve hypoxia, thereby improving the efficacy of chemotherapy (Leuci et al., 2016). It has been suggested that the use of necroptosis-based chemotherapy could provide a new alternative to the conventional treatment of drug-resistant COADs (Dasgupta et al., 2016). In this regard, we further explored small-molecule inhibitor drugs targeting the possible action of NRLPS in COAD, including Dasatinib, Imatinib, Nilotinib, Pazopanib, PLX4720. Pazopanib could target RIPK1 to act as a cytostatic inhibitor of necroptosis (Fauster et al., 2015), while the c-Src inhibitor Dasatinib could enhance necroptosis in paclitaxel-treated lung adenocarcinoma cells (Diao et al., 2016). Therefore, it should be possible to predict the effectiveness of chemotherapy and help to tailor treatment for patients with COAD according to their individual characteristics with our risk model.

Despite the positive results of the study, some limitations remain. To begin with, the risk signature is derived from the TCGA public databases which, while comprehensive, do not yet include data from new clinical samples for further validation. Aside from that, the sample size is not large enough, and the qRT-PCR is only used to validate NRLs expression in tissue samples and cell lines. It will be necessary to perform further functional studies in order to gain a better understanding of the mechanistic pathways that underlie NRLs actions. Lastly, it is notable that despite this study’s promising performance in predicting prognosis, as a retrospective study, it needs to be validated by a multicenter prospective clinical trial.

## Conclusion

The present study systematically describes an NRLPS constructed for COAD and discusses the potential functions and clinical indications. In addition, the model has also been shown to have independent prognostic value, as well as good sensitivity and reliability, which can help to predict the survival rate of COAD patients, as well as assist in elucidating immune landscape and the possible pathogenesis of COAD. Furthermore, the prediction model also provides the ability to identify COAD patients who are likely to respond well to immunotherapy and chemotherapy as well. A deeper understanding of the underlying mechanisms of this risk signature is necessary to facilitate individualized treatment of patients with COAD.

## Figures and Tables

**Table:** 

**lncRNAs**	Long noncoding RNAs
**NRLs**	Necroptosis-related long noncoding RNAs
**COAD**	Colon adenocarcinoma
**EMT**	Epithelial-mesenchymal transition
**NRLPS**	NRL prognostic signature
**TSIs**	Tumor stemness indices
**ICGs**	Immune checkpoint genes
**KEGG**	Kyoto Encyclopedia of Genes and Genomes
**LASSO**	Least absolute shrinkage and selection operator
**qRT-PCR**	Quantitative reverse transcription polymerase chain reaction
**ICGs**	Immune checkpoint genes
**GSEA**	Gene Set Enrichment Analysis
**CRC**	Colorectal cancer
**EOCRC**	Early-onset colorectal cancer
**EGFR**	Epidermal growth factor receptor
**PCD**	Programmed cell death
**COPDC**	Hronic obstructive pulmonary disease
**MLKL**	Mixed-lineagekinasedomain-like protein
**RIPK3**	Receptor-interacting serine/threonine-protein kinase 3
**DAMPs**	Damage-associated molecular patterns
**TME**	Tumor microenvironment
**TILs**	Tumor-infiltrating lymphocytes
**ICIs**	Immune checkpoint inhibitors
**TCGA**	The Cancer Genome Atlas
**CNV**	Copy number variation
**NRGs**	Necroptosis-related genes
**GO**	Gene Ontology
**BP**	Biological processes
**CC**	Cellular components
**MF**	Molecular functions
**OS**	Overall survival
**ROC**	Receiver operating characteristic
**AUC**	Area under the curve
**PCA**	Principal component analysis
**GSEA**	Gene Set Enrichment Analysis
**FDR**	False discovery rate
**ssGSEA**	Single-sample gene set enrichment analysis
**ICGs**	Immune checkpoint genes
**TMB**	Tumor mutation burden
**GDSC**	Genomics of Drug Sensitivity in Cancer
**DEGs**	Differential Expressed Genes
**SNP**	Single Nucleotide Polymorphisms
**SNV**	Single Nucleotide Variants
**IC50**	Half maximal inhibitory concentration
**CSCs**	Cancer stem cells
**ICD**	Immunogenic cell death
**MDR**	Multiple drug resistance
**Smo**	Smoothened

## Data Availability

The datasets presented in this study can be found in online repositories. The names of the repository/repositories and accession number(s) can be found in the article/Supplementary Material.
